# Problem Solving As a Sufficient Condition of the Creative Process: A Case for Closer Cooperation of Creativity Research and Problem Solving Research

**DOI:** 10.3389/fpsyg.2016.00488

**Published:** 2016-03-31

**Authors:** Lena Wimmer

**Affiliations:** Department of Psychology, University of Duisburg-EssenEssen, Germany

**Keywords:** creativity, creative process, problem solving, little c, P-creativity

In this paper, I will question the traditional definition according to which the creative process is the solving of especially complex problems. After a brief introduction, I aim to show that every instance of successful problem solving constitutes a creative process which results in a creative product. In this view, the conventionally assumed relationship of creativity and problem solving is reversed, for creativity is not regarded as a special case of problem solving but (successful) problem solving is regarded as creativity.

Creativity is considered one of the most important human competencies. It is the basis for extraordinary achievements in the arts and sciences, and enables people to adapt to changing demands (Baas et al., 2015). As creativity is among the currently most valued and desired abilities, researchers try to understand how it works and how it can be promoted. Research in this field is traditionally divided into the four strands creative person, creative process, creative press, and creative product, which Rhodes (1961) terms the “Four Ps” of creativity. Recently, creative persuasion, and creative potential have evolved as further areas of interest (Runco and Kim, 2011). The creative product takes a superior role among these strands insofar as all other lines of creativity research depend on the creative product (Groeben, 2013b): A creative person is thought of as someone who often comes up with creative ideas, i.e., someone who generates many creative products. Likewise, the creative process is usually defined as comprising all mental and behavioral events by which a person fabricates a creative product. Creative press refers to the properties of an environment which fosters or impairs the generation of a creative product. Creative persuasion is the extent to which the perception or use of a creative product changes recipients' beliefs. And finally, the creative potential is the capacity of a person to generate many creative products. Consequently, although this essay deals with the creative process, the creative product must also be kept in mind. The creative product is commonly defined as follows: A product is creative, if it is both novel and useful. The combination of novelty and usefulness is sometimes called *effective novelty* (Cropley, 2011).

While in earlier times, especially around 1800, only “real geniuses” were considered capable of generating creative products, creativity research has been dominated by a general democratization from about 1950 onwards (Groeben, 2013a). Nowadays it is commonly assumed that, in principle, everyone can be creative, albeit not to the same degree, as there will always be differences between subjects regarding the frequency and quality of creative products they generate (Cropley, 2011; Groeben, 2013a). The democratization of creativity necessitated a reconsideration of the definition of the creative product. Up to that point, it was understood that only “absolutely” novel products, i.e., ideas new to everyone in a society or even in the world, meet the requirements of a creative product. But with the change in perspective, this definition had to be extended to include products which are not necessarily new to all members of society but only to some, or in the extreme case, only to the individual who created the product. In line with this reasoning, Boden (1994) distinguishes between historical novelty as contributing to historical creativity (“H-creativity”) and personal novelty as contributing to personal creativity (“P-creativity”). Likewise, the view which deals exclusively with socially relevant achievements and the perspective that includes personally relevant achievements are often differentiated and symbolized as *Big C* and *little c*, respectively (Kaufman and Beghetto, 2009).

Having introduced some important concepts, I will now turn to the gist of this essay: the creative process. Various models conceive of the creative process as consisting of a series of phases, a notion which is often traced back to Wallas (1926). Most of the models include the four stages preparation, incubation, illumination, and verification. Preparation is a stage of intense conscious work during which information is looked for and a problem is formulated. During incubation, the problem is not consciously dealt with. Instead, unconscious processes can be at work and combine relevant pieces of information which were gathered during preparation. Illumination is characterized by a sudden insight into the solution of the problem, often referred to as “Aha!” experience (Kim, 2009). The verification stage is required to check and elaborate the solution. Since Wallas' proposition, many conceptualizations of the creative process have been put forward (e.g., Suler, 1980; Rothenberg, 1996; Simonton, 2003; Nijstad et al., 2010; Allen and Thomas, 2011). In what follows, I will comment on the widely held assumption that the creative process is a special form of problem solving, which is also referred to as creative problem solving (e.g., Treffinger and Parnes, 1979; Carson and Runco, 1999). More precisely, my deliberations will focus on a specific version of this hypothesis according to which the creative process is the solving of complex/dynamic problems (overview: Groeben, 2013b).

Before contesting this view, it is necessary to outline some basic assumptions which are held in problem solving research. This field of research typically makes a distinction between problems and tasks, so as to clarify its subject matter. According to a classic definition, a problem “arises if a living creature has a goal but does not know how this goal is to be reached” (Duncker, 1945, p. 1). Accordingly, the subject cannot achieve the desired goal simply by acting but needs to have recourse to thinking. If, on the other hand, a person wants to achieve a goal and is in principle familiar with steps leading toward that goal, then the person is dealing with a task as opposed to a problem (Groeben, 2013b). Whether or not a desired aim constitutes a problem depends, among other things, on the subject's skills (Funke, 2003). Solving the term 239–14, for instance, constitutes a (very easy) task for a vast majority of adults: Even if they cannot retrieve the solution directly from memory, they know the rules of subtraction, and these rules can be immediately applied in order to arrive at the solution. For preschoolers, however, this calculation constitutes a (most likely unsolvable) problem, because most of them do not know how to subtract multi-digit numbers. Hence, solving tasks involves procedures which are generally known to the subject, but solving problems involves procedures which are generally not known and therefore novel to the subject. Apart from the distinction between tasks and problems, problem solving researchers distinguish between different types of problems. Among these distinctions the one between simple/static and complex/dynamic^1^ problems is of special importance to creativity research, because the creative process is often conceived of as the solving of complex problems. Although the properties of these and other types of problems are quite explicitly described in the literature, it is as yet unclear to what extent differences in problem characteristics correspond to differences in the solution process (Funke, 2006). Therefore we still do not know which cognitive processes are involved in solving simple vs. complex problems, and even less whether the solution of complex problems, for instance, always requires more complex cognition than the solution of simple problems (Funke, 2010). This can be illustrated with two examples: In the Travelling Salesman Problem (TSP), a list of locations and the distances between each pair of locations are given. The aim is to find the shortest route that visits each location exactly once and returns to the starting point. Although the TSP meets the criteria of a static problem, it seems to require very complex cognitive processes, for no algorithm has been found yet which always yields the optimal solution. On the other hand, the handling of continually developing and advancing technical devices, such as smartphones, computer systems, or vending machines, is classified as dynamic problem solving (Greiff et al., 2012). Such problems, however, are often—albeit not always—solved quite quickly and without greater efforts as part of an everyday routine, which suggests that their solution does not necessarily involve very complex procedures.

In what way, then, does the creative process resemble problem solving? At the beginning of a creative process, there is always some kind of deficiency, for which the subject does not know a remedy. Thus, the creative process starts with a problem, and it is commonly accepted that a creative product is generated by means of problem solving. However, if one bears in mind that the understanding of creativity has been generally democratized, certain objections can be put forward against the view that only the solution of particularly difficult or complex problems may lead to creative products.

As elaborated above, the differentiation between simple and complex problems has proven beneficial for determining the characteristics of these two problem types. However, this distinction does not seem to help identifying the cognitive and behavioral processes with which various problems are solved—and this is exactly what one aims at when examining the creative process. If the analysis of the creative process is restricted to complex problem solving, which appears to be common practice in contemporary creativity research, problem types which at first glance do not seem to involve complex cognitive processes and hence do not seem to imply creativity may be excluded from the analysis, although their solution may in fact require a lot of creativity. If one follows this line of argument, the assumption that the creative process is the solution of complex problems needs to be modified and the creative process needs to be regarded as problem solving. In other words, problem solving has to be considered a necessary condition of the creative process.

In my view, however, this modified notion of the creative process can be challenged as well, and we even have to go one step further. For, if the above question “In what way does the creative process resemble problem solving?” is reversed to “In what way does problem solving resemble creativity?,” it becomes obvious that every instance of successful problem solving implies a creative process, given that one follows the democratization of creativity and admits P-creativity: The solution of a problem must be new to the solver, because if the subject had been acquainted with the solution from the outset, it would not be a problem, but a task (see above). Thus, every solution of a problem is novel (to the subject). In addition, the solution of a problem is always useful; otherwise the problem would not have been successfully solved. This means that all problem solutions meet the criterion of effective novelty and hence are creative products. And as the procedures yielding a creative product are said to constitute a creative process, successful problem solving can be regarded as a creative process. It can be deducted, then, that the conventionally held relation between creativity and problem solving is reversed, in that the creative process does not constitute a special case of problem solving, but successful problem solving can be regarded as a sufficient condition of the creative process.

What conclusions can be drawn from this line of argument? The first consequence is that the view of the creative process must be changed in the direction of further democratization and demystification. At present, it is accepted that the creative process does not involve any mystic or godlike properties (Kim, 2009), but, following the above deliberations, one would have to go further and regard the creative process as no more than regular problem solving. Hence, designations such as creative problem solving appear to contain a redundancy. Second, a closer cooperation of creativity research and problem solving research suggests itself. If the creative process is the same as problem solving, then scientific investigations should examine it as such, using appropriate methods, i.e., the methods of problem solving research. This does not mean that we can now dispense with creativity research altogether. Rather, one should bear in mind what both research traditions have in common and how each could benefit from the other. Third, once a new perspective of the creative product and the creative process has been discussed, this may also have ramifications for the two remaining strands of creativity research, the creative person and creative press. If the creative product and process can be seen from the perspective of problem solving research, could this also apply to the creative person and the creative press? Can creative persons be viewed as sharing relevant traits with persons who like to solve problems and often do so? Can the creative press be compared to an environment which promotes problem solving? I would like to suggest that creativity research could obtain more differentiated knowledge of its field if it investigated to what extent creativity is different from or identical with problem solving.

## Author contributions

LW contributed to the work in the following ways: conception of the work; drafting and revising it critically for intellectual content; approval of the final version to be published; agreement to be accountable for all aspects of the work in ensuring that questions related to the accuracy or integrity of any part of the work are appropriately investigated and resolved.

### Conflict of interest statement

The author declares that the research was conducted in the absence of any commercial or financial relationships that could be construed as a potential conflict of interest.

## Footnotes

^1^Funke (2003) suggests that simple problems should be termed static and complex problems dynamic. Hence, the terms simple/static problem and complex/dynamic problem are used interchangeably in this essay.
